# The Lungs

**Published:** 1872-05

**Authors:** 


### THE LUNGS.

In one week in February there were four hundred and eighty-six deaths in Philadelphia, one quarter of which were from diseases of the throat and lungs; one out of every eight dying of consumption, in one of the healthiest cities of the Union.

It admits of question whether consumption is ever inherited. Louis of Paris, the best authority of the age in such matters, states that the deaths from consumption of persons whose parents were consumptive, were not greater in proportion than those whose parents had not died of consumption. These things point indisputably to the fact that consumption is an acquired disease, an avoidable disease, a disease brought upon the individual by his own conduct — avoidable, with a moderate amount of information as to the causes of consumption: bad air in bedrooms for a third of one's entire existence, to begin with; bad feeding, which comes from bad cookery, even if the food was the best in the world before it went to the kitchen; irregular and insufficient sleep; neglect of out-door activities; inattention to the bodily functions; the amazing consumption of patent medicines and medicinal waters; thin shoes; insufficient clothing; reckless exposures to changes of temperature; and injudicious changes of clothing to what is less warm, even on a warm day.

The foundation of three fourths of all cases of common consumption of the lungs is laid in the teens, and yet not one family in one hundred in the United States, takes any publication like this, the object of which is to disseminate information as to the best means of taking care of the health without medicine.

And yet in these same families, at least in many of them, a large amount of money is spent for novels, for newspapers with trashy stories, and magazines of a like character. Let thoughtful parents consider this.

---

Rules for May. — Warm weather is coming; we need less internal heat; and as this internal heat comes from the food we eat, we do not need as much food: hence, like a watchful mother, nature takes away our appetite, so that we may not eat so much as before, to burn us with fever.

